# Massive Burns Versus Less Extensive Burns in Children: Clinical Outcomes and Independent Predictors of Mortality

**DOI:** 10.3390/children13101364

**Published:** 2026-10-08

**Authors:** Zeliha Akış Yıldız, Aslı Nur Abay, Vildan Selin Cayan, Süleyman Arif Bostancı, Elif Emel Erten, Ahmet Ertürk, Can İhsan Öztorun, Sabri Demir

**Affiliations:** Department of Pediatric Surgery, Pediatric Burn Center, Children’s Hospital, Ankara Bilkent City Hospital, Ankara 06800, Türkiye; aslinur.abay@sbu.edu.tr (A.N.A.); vildanselin.sahin@saglik.gov.tr (V.S.C.); suleymanarifbostanci@aybu.edu.tr (S.A.B.); elifemel.erten@saglik.gov.tr (E.E.E.); ahmeterturk@aybu.edu.tr (A.E.); cioztorun@aybu.edu.tr (C.İ.Ö.); sabri.demir1@saglik.gov.tr (S.D.)

**Keywords:** pediatric burns, massive burns, mortality, intubation

## Abstract

**Highlights:**

**What are the main findings?**

- Mortality increased markedly in pediatric patients with burns involving ≥50% of the total body surface area (TBSA), and flame burns and inhalation injury were significantly more frequent in this group.
- Intubation and burn extent ≥50% TBSA were independently associated with mortality in multivariable analysis.

**What are the implications of the main findings?**

- Given the limited pediatric data specifically focusing on massive burns, this study adds clinically relevant evidence on the characteristics and outcomes of this high-risk population.
- Burn extent and the need for intubation may provide simple and clinically useful indicators for early mortality risk stratification in children with massive burns.

**Abstract:**

Background/Objectives: Massive burns are associated with substantial morbidity and mortality in children. Data comparing pediatric burns of different severities and identifying mortality predictors remain limited. This study aimed to compare the clinical characteristics and outcomes of children with burns involving 20–49% and ≥50% total body surface area (TBSA) and to identify independent predictors of mortality. Methods: This retrospective cohort study included children with burns involving ≥20% TBSA treated at a national referral pediatric burn center. Patients were categorized as 20–49% TBSA or ≥50% TBSA. Demographic characteristics, burn etiology, inhalation injury, intubation, intensive care requirements, length of hospital stay, pBaux score, and mortality were analyzed. Multivariable logistic regression was used to identify mortality predictors. Results: Among 260 children, 206 had burns involving 20–49% TBSA and 54 had burns involving ≥50% TBSA. Scald burns predominated in the 20–49% TBSA group, whereas flame burns were more frequent in the ≥50% TBSA group. Inhalation injury and intubation were also more common in the ≥50% TBSA group. Mortality was significantly higher in patients with ≥50% TBSA burns (29.6% vs. 3.4%; *p* < 0.001). Burn extent ≥50% TBSA (OR 14.30) and intubation (OR 15.59) independently predicted mortality. Conclusions: Massive pediatric burns are associated with markedly worse outcomes. Burn extent ≥ 50% TBSA and intubation may help identify children at particularly high risk of mortality.

## 1. Introduction

Childhood burns are a common cause of injury and mortality in children, while also imposing substantial socioeconomic burdens on affected families and healthcare systems. Although burns are largely preventable, they remain an important cause of morbidity and mortality in low- and middle-income countries. In contrast, high-income countries have achieved substantial reductions in burn-related mortality through advances in prevention strategies, specialized burn care, intensive care support, and surgical management. However, limited access to specialized burn centers, delayed presentation, inadequate resuscitation, restricted critical care resources, and limited surgical capacity continue to adversely affect outcomes in many low- and middle-income settings. Although burns involving ≥50% of the total body surface area (TBSA) are relatively uncommon in pediatric populations, they are associated with substantial morbidity and mortality and represent a particularly vulnerable subgroup whose outcomes may vary considerably across centers and healthcare systems [1,2,3,4,5,6].

At a panel during the European Burn Association (EBA) 2025 congress, it was reported that the number of pediatric burn patients with a TBSA ≥ 50% has decreased in many European countries, to the point where such cases occur less than once a year. While this is obviously a positive development, there are concerns regarding the training of healthcare professionals to deal with pediatric cases of massive burns. Nevertheless, a significant number of pediatric massive burn cases are still encountered in Türkiye each year [7].

In children with severe burns, it has been shown that mortality is determined by the extent of the burn, presence or absence of inhalation injury, need for mechanical ventilation, and infection. Despite advances in burn treatment, mortality remains high for pediatric burns exceeding 50% of TBSA [6,8].

Although numerous studies have investigated the epidemiology, outcomes, and prevention strategies associated with burn injuries, data concerning pediatric patients with burns covering 50% or more of TBSA are limited. Most data are from high-income countries and pertain to adults [6,8,9]. Given this background, we aimed to contribute to burn prevention strategies by obtaining an overview of the clinical characteristics, treatment, and outcomes of patients with burns covering 50% or more of their body surface area who were treated at our tertiary pediatric burn center.

## 2. Materials and Methods

This study was conducted at the Pediatric Burn Center of Ankara Bilkent City Hospital, a tertiary referral center, following the receipt of ethics committee approval (TABED 1-26-2276; 25 February 2026). Patients aged <18 years who received inpatient treatment at our hospital between 1 August 2019 and 31 December 2025, and who had a total burn area of ≥20% TBSA, were included. Of the 1542 patients, 1282 were excluded because their burn area was less than 20% of TBSA. Data regarding age, burn etiology, burn mechanism, inhalation injury, need for mechanical ventilation, length of hospital stay (LOS), and mortality were retrospectively reviewed from the medical records of 260 patients with TBSA ≥ 20%. In addition, because the main study cohort excluded patients with smaller burns, the medical records of infants and toddlers treated during the same period were separately reviewed to identify documented cases of non-accidental injury (NAI), including those with burns involving <20% TBSA.

Burn depth was assessed clinically by experienced burn surgeons based on wound appearance, color, capillary refill, moisture, blister formation, pain response, and tissue characteristics. Serial wound assessments were performed during hospitalization when necessary to account for changes in burn depth over time. No device-based assessment method, such as laser Doppler imaging, was routinely used. TBSA was calculated using the Lund–Browder chart [10,11]. Surgical treatment was individualized according to burn depth, extent, anatomical involvement, and clinical status. Procedures included escharotomy, necrectomy, surgical debridement, fasciotomy, split-thickness skin grafting, flap reconstruction, and amputation when clinically indicated. The patients were divided into two groups based on TBSA: less extensive burns (20–49%; n = 206) and massive burns (TBSA ≥ 50%; n = 54). The two groups were compared in terms of age, sex, place of residence, time to admission to the burn center, need for grafting, development of contractures and/or hypertrophic scars, occurrence of amputation, inhalation injury, need for intubation, pediatric Baux (pBaux) score, length of hospital stay, and mortality. The pBaux score, a modified version of the Baux score incorporating inhalation injury, was calculated as TBSA (%) − age + (18 × R), where R = 1 in the presence of inhalation injury and R = 0 otherwise [12,13]. Burn etiology (scald, fire/flame, electrical, other) and mechanism (related to household cooking, traditional outdoor cooking, solid-fuel heating, flammable substance, explosion, high-voltage electrical burns, and others) were compared according to the burn surface area.

Subsequently, the patients were divided into two groups—survivors and non-survivors—and these groups were compared in terms of age, sex, inhalation injury, need for intubation, pBaux score, TBSA, and LOS. The diagnosis of inhalation injury was established by bronchoscopy in patients of all ages in whom inhalation injury was clinically suspected. The need for mechanical ventilation was determined in accordance with standard airway protocols. Additional diagnostic investigations were performed according to the clinical condition of each patient. Chest radiography was obtained in patients with suspected respiratory involvement or inhalation injury, while arterial blood gas analysis and other imaging studies were performed when clinically indicated.

The distribution of TBSA groups across months was also examined to evaluate temporal variations in burn severity. Kaplan–Meier survival analysis was used to estimate survival probability over time and to compare survival between patients with TBSA 20–49% and TBSA ≥ 50%. Patients who survived until the end of follow-up were considered censored. Differences between survival curves were evaluated using the log-rank (Mantel–Cox) test. Multivariate logistic regression analysis was performed to identify independent predictors of mortality.

Statistical analyses were performed using the Statistical Package for the Social Sciences (SPSS) software version 21.0 (IBM Corp., Armonk, NY, USA). Continuous variables were presented as mean ± standard deviation. The continuous variables (age, LOS, and pBaux score) were first examined using the Kolmogorov–Smirnov test, a goodness-of-fit test used to assess whether the distribution of the data deviated significantly from a normal distribution. Parametric or non-parametric statistical tests were selected according to the distribution of the data. Upon confirming that all of the data followed a normal distribution, differences between groups were investigated using Student’s *t*-test. Categorical variables are expressed as numbers and percentages. Differences between groups were investigated using the chi-square test. When a difference was detected among more than two categorical groups, pairwise comparisons were performed using Bonferroni correction to identify which specific groups differed. Survival analyses were performed using the Kaplan–Meier method. The differences between the survival curves were evaluated using the log-rank (Mantel–Cox) test. Multivariate logistic regression analysis was performed to identify variables independently affecting mortality. The variables were determined based on clinical relevance and univariate analysis results. Adjusted odds ratios (ORs) were calculated along with their confidence intervals (CIs). Statistical significance was set at *p* < 0.05.

## 3. Results

### 3.1. Demographic and Burn Characteristics

The 260 pediatric burn patients with ≥20% TBSA treated at our burn center between 1 August 2019, and 31 December 2025 ranged in age from 40 days to 17 years, with a mean age of 5.53 ± 5.14 years. Regarding sex, 91 (35%) were female and 169 (65%) were male. The mean age of patients in the less extensive burns group was 4.88 ± 4.68, while that in the massive burn group was 8.01 ± 6.04. Massive burns were more common in older children (*p* < 0.001). Although males predominated in both groups, no difference in the sex ratio was observed between the groups.

No difference was found when comparing the regions from which the patients originated (*p* = 0.507). However, owing to the hospital’s status as a referral institution, the number of patients from outside Ankara was higher than that of patients from Ankara.

### 3.2. Clinical Outcomes According to Burn Extent

The proportion of patients presenting to the hospital within the first 24 h after the burn injury was 61.9%, and this variable showed no difference between the groups. Post-treatment complications included contracture formation and/or hypertrophic scarring in 150 patients (57.7%) and amputation in 12 patients (4.6%). Contracture/hypertrophic scar formation was observed in 116 patients (56.3%) in the TBSA 20–49% group and 34 patients (63.0%) in the TBSA ≥ 50% group, whereas amputation was required in 8 (3.9%) and 4 (7.4%) patients, respectively. The grafting rate was 65.5% for less extensive burns and 75.9% for massive burn cases, which were also not significantly different. Similarly, no significant intergroup differences were found in the rate of hypertrophic scarring, contractures, or amputation (Table 1).

Both inhalation injury and the need for intubation were more common in patients with massive burns (both *p* < 0.001). Similarly, the pBaux score and LOS were higher in patients with massive burns (both *p* < 0.001). The mortality rate was significantly higher in patients with massive burns than in the less extensive burns group, at 29.6% vs. 3.4% (*p* < 0.001).

Mortality showed a marked increase with greater burn extent, particularly above 60% TBSA, reaching 100% in patients with 90–100% TBSA involvement (Table 2).

No cases of non-accidental injury were identified among the 260 patients included in the main study cohort, all of whom had burns involving ≥20% TBSA. In the additional review of 119 infants and toddlers treated during the same study period, one case of NAI was identified (0.8%); this patient had a burn involving <20% TBSA and was therefore not included in the main study cohort.

Regarding the mechanism of injury, household cooking was the leading cause of less extensive burns, whereas it was less frequently observed in massive burns (*p* < 0.001). Traditional outdoor cooking-related and solid-fuel heating-related burns were more common in patients with massive burns (*p* < 0.001). Scald burns were more common in the less extensive burns patient group (*p* < 0.001), whereas flame burns were more common in the massive burn patient group (*p* = 0.002). No differences were observed between the groups regarding the rates of burns caused by igniters, burns resulting from explosions, or electrical burns (Table 3).

The monthly distribution of admissions due to burns is shown in Figure 1. A peak in admissions of patients with TBSA ≥ 50% occurred during the late summer and early fall. Conversely, the number of admitted patients with TBSA of 20–49% exhibited less fluctuation across the course of a year. Although such cases were more common during the late summer and early autumn, no seasonal distribution was observed.

### 3.3. Comparison of Survivors and Non-Survivors

No differences in sex and age were detected between surviving and non-surviving patients in the massive group. Although the difference in the rates of inhalation injury was not statistically significant, this rate was higher in non-survivors (50% vs. 28.9%, *p* = 0.130). Meanwhile, the intubation rate was higher in non-survivors (*p* < 0.001), as was the pBaux score (*p* = 0.002). Similarly, the TBSA was significantly larger in non-survivors than in survivors (*p* = 0.003). As expected, LOS showed the opposite trend (*p* = 0.005) (Table 4).

### 3.4. Independent Predictors of Mortality

In the multivariate logistic regression analysis, intubation, TBSA ≥ 50%, and LOS were independent predictors of mortality (Table 5). Intubation was associated with a significantly higher risk of mortality (OR: 15.59, 95% CI: 3.98–61.04, *p* < 0.001). Similarly, TBSA ≥ 50% was associated with high mortality (OR: 14.30, 95% CI: 4.11–49.83, *p* < 0.001). Furthermore, LOS independently increased the risk of mortality (OR: 1.04, 95% CI: 1.01–1.07, *p* = 0.003). However, inhalation injury was not found to be a determinant of mortality (OR: 1.28, 95% CI: 0.29–5.55, *p* = 0.745). While the need for intubation and a large burn area were found to be the strongest predictors of mortality, prolonged LOS also increased the mortality risk.

### 3.5. Survival Analysis

Kaplan–Meier survival analysis revealed a higher mortality rate in patients with massive burns. The survival curves did not subsequently converge and remained clearly separated at 6 months after the burn injury and beyond. Follow-up data showed a higher probability of survival in the 20–49% TBSA group (Figure 2). Consistent with these findings, statistical significance in this difference in survival was observed in the log-rank (Mantel–Cox) test (*p* < 0.001).

## 4. Discussion

The incidence of massive burns has decreased in developed countries, leading to low- and middle-income countries now accounting for 90% of the global burn burden. Compared with the rates in Western Europe, for example, high rates of severe burns are still reported in developing countries such as Turkiye [7,14].

In our study, a TBSA of ≥50% and the need for intubation in pediatric patients with burns were identified as the strongest independent predictors of mortality. This highlights the critical role of burn severity in determining survival outcomes. As in previous studies, burn size was the most important factor determining mortality [15,16,17].

Although not statistically significant, grafting, development of hypertrophic scars and contractures, and amputation were observed more frequently in patients with ≥50% of TBSA. As noted in the literature, the presence of deeper tissue burns in extensive burn injuries leads to long-term complications owing to the prolonged healing period [17].

In our study, the clinical characteristics of pediatric patients with massive burns were evaluated based on TBSA and survival. Accordingly, greater TBSA, the need for intubation, the presence of inhalation injury, and higher pBaux score adversely affected the prognosis. In terms of survival, the need for intubation in cases with ≥50% TBSA was significantly higher than that in the non-survival group. This suggests that the need for mechanical ventilation is a determinant of poor prognosis in patients with massive burns. Furthermore, in the regression analysis, intubation was shown to increase the risk of mortality more than 15-fold. These findings are in line with previous studies [16]. In our study, although the rate of inhalation injury was higher in patients who died, the difference was not statistically significant. In addition, inhalation injury was significantly more common in patients with massive burns than in those with less extensive burns. Consistent with the literature, higher TBSA and Baux scores were associated with mortality [12,13,18].

Although mortality increased substantially with increasing TBSA, our findings do not support a specific burn-size threshold beyond which treatment could be considered futile. Survival was still observed among patients with 70–89% TBSA burns, whereas the number of patients with 90–100% TBSA burns was too small to define this range as uniformly non-survivable. Moreover, survival after burns exceeding 90% TBSA has been reported in other pediatric series, further indicating that burn size alone should not be used to define the futility of care [15]. Therefore, decisions regarding treatment limitation should consider not only TBSA but also additional factors such as inhalation injury, ventilatory requirement, age, and overall physiological status.

Although LOS is typically short for non-survivors, the duration of hospitalization increases with increasing extent of burns in surviving patients. However, although a prolonged stay may reflect increased disease severity, it can also be associated with survivorship bias. Specifically, while patients who survive longer may accumulate complications over time, LOS can be particularly short for the most severe patients given that they often die at an early stage. Meanwhile, in this study, patients with a burn size of ≥50% TBSA had longer hospital stays than those with 20–50% TBSA because of the need for repeated surgeries, wound care, and rehabilitation. Consistent with the literature, and owing to the complexity of treatment, survivors of massive burns require prolonged hospitalization [16,19,20].

The mean age was higher in cases of massive burns than in those with less extensive burns. Scald burns were more common in younger age groups, whereas flame burns and high-energy trauma were more frequently observed in older children and adolescents. Flame burns have been identified as the most significant cause of severe burns and the primary reason for associated morbidity and mortality [16,21], which aligns with our findings.

Only a small proportion of patients treated in our study were from Ankara, where our hospital is located. Most instead transferred from other provinces in Türkiye and abroad. Patients from abroad were predominantly from neighboring Syria, likely reflecting the ongoing conflict in the country. Such patients with massive burns are typically referred for complex, multidisciplinary care at our tertiary referral hospital [22].

The high incidence of massive burns in developing countries has been attributed to cultural, socioeconomic, and environmental factors. As in many developing countries, open fires are widely used for cooking and heating in Türkiye. They are traditionally used to prepare food for the winter, especially in late summer and early autumn. These foods include cheese, tomato paste, çökelek (dry curd cheese), marmalade, jam, and preserves, which are typically prepared in large cauldrons in the garden. Bread and meals can also be cooked over a fire in the garden. While mothers are occupied with this task, children often also play in the garden. In these circumstances, children can suffer burns from accidentally tipping over the cauldron or falling into it or the fire. There are also patients who sustain burns after falling into a fire. These customs contribute to the incidence of scalds and flame burns in Turkish children and may also explain the seasonal variations observed in massive burns [23,24].

In terms of heating-related causes of burn cases encountered in Türkiye, wood-burning stoves used during the winter months, as well as diesel-fueled stoves used by Syrian patients, also cause severe burns. Furthermore, similar to the situation in other developing countries, poverty-related practices in Türkiye, such as cooking meals, preparing hot beverages, and heating bathwater on stoves or heaters that are also used to warm living areas, can lead to serious burns [25].

The Kaplan–Meier survival curve for the ≥50% TBSA group in this study showed a decline in survival that began early and progressively worsened over time. This suggests that mortality is both higher and occurs earlier in patients with massive burns. The systemic effects of extensive burns may result from inhalation injury, sepsis, and multiple organ failure. This demonstrates that the early phase of massive burns constitutes a “high-risk window” and that an aggressive, multidisciplinary approach is critically important during this period. In contrast, survival probabilities for patients with 20–49% TBSA remained relatively stable over time, indicating more effective physiological compensation and a better response to treatment. This demonstrates that TBSA is not only associated with increased mortality, as shown in the study by Wardhana et al., but also significantly influences the timing of death [26,27].

In our study, multivariate analysis findings indicated that mortality in pediatric burn cases was primarily determined by burn extent and the need for respiratory support. These results support the use of TBSA and intubation status as key components in early risk stratification models and highlight the importance of aggressive and timely critical care management in patients with extensive burns and respiratory failure.

Mortality rates following massive pediatric burns exhibit significant variability across various healthcare environments. A recent decade-long study conducted at a specialized pediatric burn center in the United States documented a mortality rate of 25% among children with burns involving ≥50% TBSA, despite a mean burn size of 66% TBSA [6]. Similarly, another U.S. burn center documented mortality rates of <20% among children with burns covering 50–80% of the TBSA and approximately 55% for those with burns involving ≥90% TBSA. The overall mortality rate for children with burns covering ≥50% TBSA was approximately 21%, indicating significant advancements in survival rates in high-income settings [15]. Conversely, significantly poorer outcomes have been observed in resource-limited settings. In a South African pediatric burn unit, no child with burns exceeding 60% of the TBSA survived [28]. Similarly, an Indian study documented a mortality rate of 76.8% among children with burns covering 51–75% of the TBSA, with no survivors among those with 76–100% TBSA burns [29]. These findings illustrate significant variability in survival rates following extensive pediatric burns and imply that disparities in access to specialized burn care, critical care capacity, early surgical intervention, and multidisciplinary treatment may contribute to differences in outcomes across healthcare systems.

Nevertheless, in the present study, the mortality rate observed among children with massive burns was closer to those reported from high-income countries than to the substantially higher rates described in low- and middle-income settings [6,15,28,29]. This finding suggests that outcomes in our center may be approaching those achieved in well-resourced burn care systems despite the challenges associated with managing extensive pediatric burns. However, the actual incidence of massive burns is low in many European countries [7,30,31,32], while they remain a serious problem in Türkiye. While we can raise awareness and help to prevent burns through education, reducing long-term burn rates may be possible only through improvements in socioeconomic status.

Non-accidental burns represent an important concern in pediatric burn care. In a systematic review by Loos et al., the reported prevalence of non-accidental pediatric burns ranged from 0.5% to 24.6%. Similarly, a study from the United Kingdom reported a prevalence of 15.8%, indicating that non-accidental burns constitute a clinically relevant proportion of pediatric burn injuries. In the present study, no cases of NAI were identified within the main cohort, which included only patients with burns involving ≥20% TBSA. However, an additional review of infants and toddlers treated at our center during the same study period identified one case of NAI among 119 patients (0.8%); this patient had a burn involving <20% TBSA and was therefore not included in the present study cohort. The markedly lower rate observed in our center compared with previous reports may reflect differences in patient selection, but it may also suggest possible under-recognition or under-documentation of NAI. Therefore, greater clinical vigilance is warranted, particularly in younger children, those presenting with scald or deep burns, burns involving the posterior trunk, buttocks, or genital region, and children with associated injuries such as bruising or fractures. Systematic assessment for possible abuse or neglect should be incorporated into the evaluation of pediatric burn patients, especially when the injury pattern or clinical history raises concern [33,34].

This study had a range of limitations, which should be mentioned here. First, the retrospective nature of our study may have resulted in some data being missed. Moreover, as this study focused on a single institution that acts as a referral center for pediatric burns, the proportion of more complex and severe burns was higher than at other burn centers. Specifically, burns covering less than 20% of TBSA constitute the majority of the burn patient population. Since we did not include such patients here, our mortality rate may also be higher than expected compared with the findings of studies that include all burn categories. Furthermore, the small size of our patient group with TBSA ≥ 50% may have limited the robustness of our findings. Nationwide multicenter studies should be performed to overcome this limitation.

## 5. Conclusions

Our study revealed that a large TBSA is the most important predictor of mortality and adverse clinical outcomes in pediatric patients with burns. Massive burns are associated with a higher incidence of inhalation injury, need for intubation, longer LOS, and reduced survival. While the need for intubation and TBSA ≥ 50% are independent predictors of mortality, the impact of demographic factors on prognosis is more limited.

The etiology and mechanism of burns in children significantly influence burn surface area. While less extensive burns are predominantly caused by scalding, flame burns are the primary cause of massive burn injuries. Prevention strategies should focus on reducing high-energy burns, particularly those caused by fire. Morbidity and mortality in pediatric burns should be reduced through early risk analysis, intensive care, and prevention strategies targeting high-risk burn mechanisms. In addition, non-accidental burn injuries should not be overlooked, and careful assessment is particularly warranted in younger children and those with suspicious injury patterns or associated injuries.

## Figures and Tables

**Figure 1:**
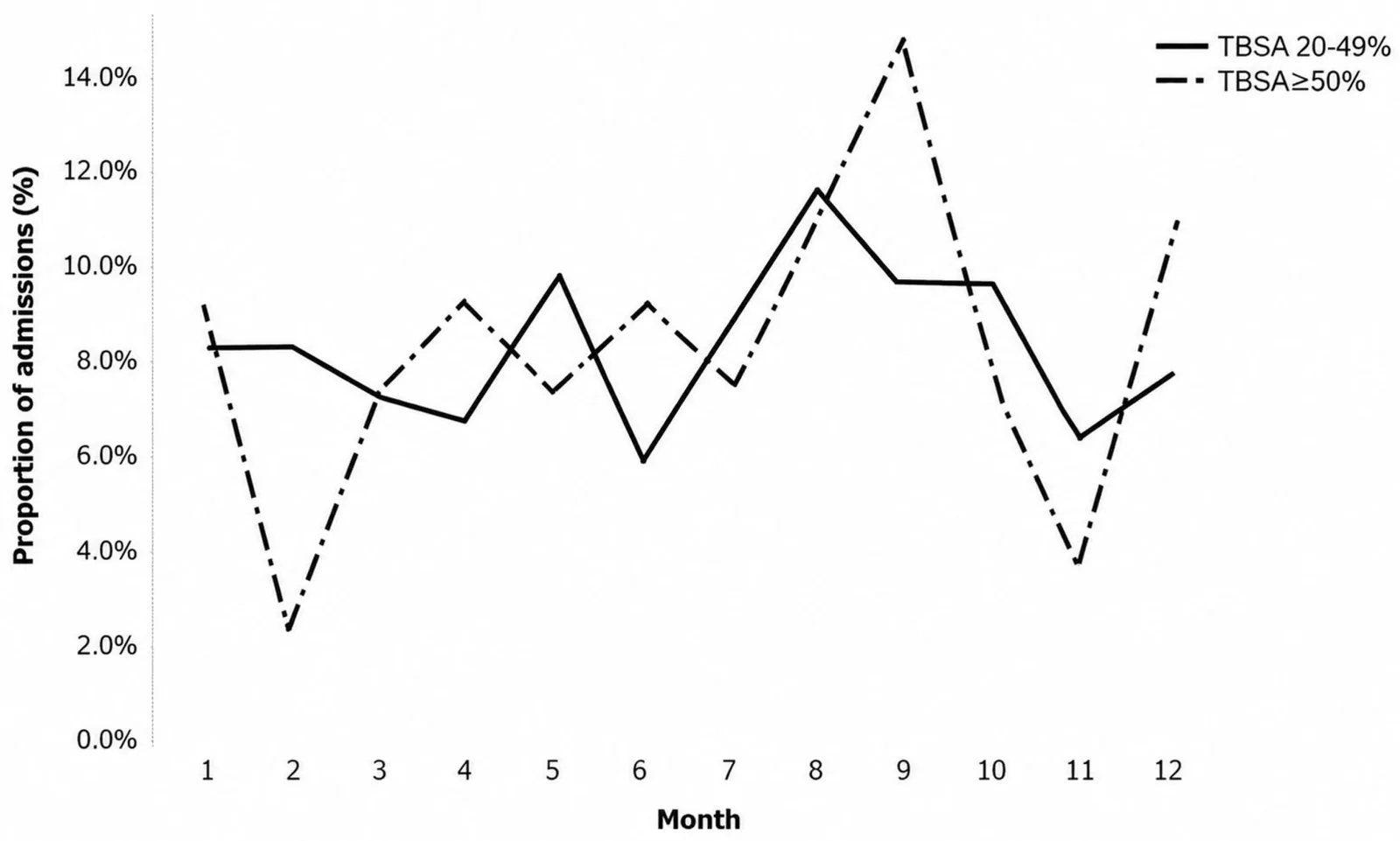
Monthly distribution of burn admissions according to TBSA group. The solid line represents patients with a total body surface area (TBSA) of 20–49%, and the dash–dot line represents patients with TBSA ≥ 50%. Values are expressed as the proportion of admissions within each burn severity group.

**Figure 2:**
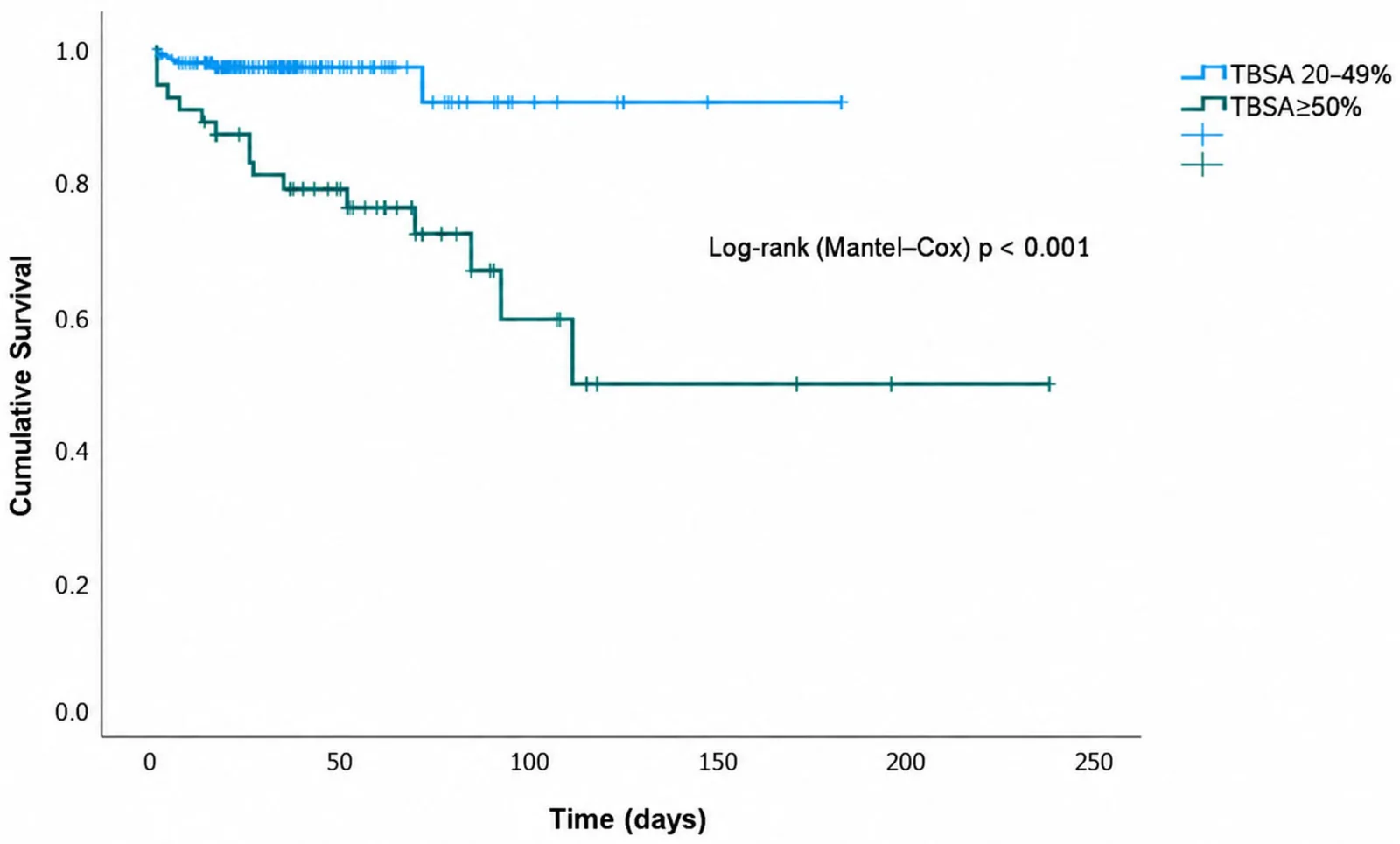
Kaplan–Meier survival curves according to TBSA group. Kaplan–Meier survival curves comparing patients with TBSA 20–49% and TBSA ≥ 50%. Patients with TBSA ≥ 50% exhibited significantly lower survival probabilities (log-rank test, *p* < 0.001).

**Table 1:** Comparison of demographic and clinical characteristics according to TBSA group.

	TBSA 20–49% (n = 206)	TBSA ≥ 50% (n = 54)	Total (n = 260)	*p* Value
Age (years), mean ± SD	4.88 ± 4.68	8.01 ± 6.04	5.53 ± 5.14	<0.001
Sex, n (%)				0.770
Male	133 (64.6)	36 (66.7)	169 (65.0)	
Female	73 (35.4)	18 (33.3)	91 (35.0)	
Country of residency, n (%)				0.507
Other cities	141 (68.4)	41 (75.9)	182 (70.0)	
Ankara	30 (14.6)	5 (9.3)	35 (13.5)	
From abroad	35 (17.0)	8 (14.8)	43 (16.5)	
Hospital admission within 24 h, n (%)				0.877
Yes	127 (61.7)	34 (63)	161 (61.9)	
No	79 (38.3)	20 (37)	99 (38.1)	
Skin grafting, n (%)				0.190
Yes	133 (65.5)	41 (75.9)	174 (67.7)	
No	73 (34.5)	13 (24.1)	86 (32.3)	
Contracture formation and/or hypertrophic scar, n (%)				0.370
Yes	116 (56.3)	34 (63)	150 (57.7)	
No	90 (43.7)	20 (37)	110 (42.3)	
Amputation				0.270
Yes	8 (3.9)	4 (7.4)	12 (4.6)	
No	198 (96.1)	50 (92.6)	248 (95.4)	
Inhalation injury, n (%)				<0.001
Yes	20 (9.7)	18 (33.3)	38 (14.6)	
No	186 (90.3)	36 (66.7)	222 (85.4)	
Intubation history, n (%)				<0.001
Yes	39 (18.9)	25 (46.3)	64 (24.6)	
No	167 (81.1)	29 (53.7)	196 (75.4)	
pBAUX score, mean ± SD	27.23 ± 29.04	58.32 ± 26.37	33.68 ± 29.70	<0.001
Length of hospital stay (days), mean ± SD	36.51 ± 25.53	61.37 ± 47.08	41.67 ± 32.74	<0.001
Outcome, n (%)				<0.001
Death	7 (3.4)	16 (29.6)	23 (8.8)	
Survived	199 (96.6)	38 (70.4)	237 (91.5)	

Categorical variables were compared using the Chi-square test and continuous variables using the independent samples *t*-test as appropriate. *p* < 0.05 was considered statistically significant.

**Table 2:** Mortality according to total body surface area burned.

TBSA, %	Patients, n	Deaths, n	Mortality, %
20–29	135	2	1.5
30–39	44	4	9.1
40–49	27	1	3.7
50–59	28	4	14.3
60–69	10	3	30
70–79	11	5	45.5
80–89	3	2	66.7
90–100	2	2	100

TBSA, total body surface area. Mortality was calculated as the number of deaths divided by the total number of patients within each TBSA category.

**Table 3:** Distribution of burn etiology and mechanisms according to TBSA group.

	TBSA 20–49% (n = 206)	TBSA ≥ 50% (n = 54)	Total (n = 260)	*p* Value
Burn etiology, n (%)				
Scald	136 (66.0)	16 (29.6)	152 (58.5)	<0.001
Fire/flame	58 (28.2)	30 (55.6)	88 (33.8)	0.002
Electrical	10 (4.9)	8 (14.8)	18 (6.9)	0.080
Other *	2 (1.0)	0 (0.0)	2 (0.8)	0.921
Burn mechanism, n (%)				
Household cooking-related burns	113 (54.9)	5 (9.3)	118 (45.4)	<0.001
Traditional outdoor cooking-related burns	27 (13.1)	14 (25.9)	41 (15.8)	<0.001
Solid-fuel heating-related burns	18 (8.7)	11 (20.4)	29 (11.2)	<0.001
Flammable substance-related burns	7 (3.4)	6 (11.1)	13 (5.0)	0.091
Explosion-related burns	17 (8.3)	3 (5.6)	20 (7.7)	0.963
High-voltage electrical burns	10 (4.9)	8 (14.8)	18 (6.9)	0.005
Other **	14 (6.8)	7 (13.0)	21 (8.1)	0.343

Comparisons between TBSA groups were performed using the Chi-square test. To control for multiple comparisons, Bonferroni correction was applied; the adjusted significance level was set at *p* < 0.006 for burn etiology and *p* < 0.003 for burn mechanism analyses. Other *: lightning injuries and sunburn. Other **: lightning injuries, sunburn, and burns due to unexplained house or vehicle fires.

**Table 4:** Comparison of clinical characteristics between non-survivors and survivors among pediatric patients with TBSA ≥ 50% burns.

	Non-Survived (n = 16)	Survived (n = 38)	*p* Value
Age (years), mean ± SD	9.60 ± 6.65	7.33 ± 5.73	0.240
Sex, n (%)			0.290
Male	9 (56.3)	27 (71.1)	
Female	7 (43.8)	11 (28.9)	
Inhalation injury, n (%)			0.130
Yes	8 (50.0)	11 (28.9)	
No	8 (50.0)	27 (71.1)	
Intubation, n (%)			<0.001
Yes	13 (81.3)	12 (31.6)	
No	3 (18.7)	26 (68.4)	
pBAUX score, mean ± SD	68.69 ± 19.17	54.92 ± 11.61	0.002
TBSA, mean ± SD (%)	69.31 ± 14.40	56.32 ± 8.42	0.003
Length of hospital stay (days), mean ± SD	38.19 ± 30.79	66.94 ± 33.72	0.005

Categorical variables were compared using the χ^2^ test and continuous variables were analyzed using the independent samples *t*-test. Statistical significance was defined as a two-tailed *p* value < 0.05. TBSA denotes total body surface area, and pBaux represents the pediatric Baux score (TBSA (%) − age + (18 × R), where R = 1 in the presence of inhalation injury and R = 0 otherwise).

**Table 5:** Multivariate logistic regression analysis of mortality predictors.

	Odds Ratio (OR)	95% CI	*p* Value
Intubation	15.59	3.98–61.04	<0.001
Inhalation injury	1.28	0.29–5.55	0.745
TBSA ≥ 50%	14.30	4.11–49.83	<0.001
Length of stay (LOS)	1.04	1.01–1.07	0.003

Odds ratios (ORs) were obtained from multivariate logistic regression analysis. Variables were selected based on clinical relevance and univariate analysis. A *p* value < 0.05 was considered statistically significant.

## Data Availability

The data presented in this study are available from the corresponding author upon reasonable request. The data are not publicly available due to patient privacy and ethical restrictions.

## Abbreviations

The following abbreviations are used in this manuscript:

TBSA	Total body surface area
EBA	European Burn Association
LOS	Length of hospital stay (LOS)
